# Depression and suicidal behavior in South Asia: a systematic review and meta-analysis

**DOI:** 10.1017/gmh.2022.20

**Published:** 2022-04-01

**Authors:** S M Yasir Arafat, Tamkeen Saleem, Vikas Menon, Syeda Ayat-e-Zainab Ali, Anuradha Baminiwatta, Sujita Kumar Kar, Hasina Akter, Rakesh Singh

**Affiliations:** 1 Department of Psychiatry, Enam Medical College and Hospital, Dhaka-1340, Bangladesh; 2 Department of Psychology, International Islamic University, Islamabad-44000, Pakistan; 3 Additional Professor Department of Psychiatry, Jawaharlal Institute of Postgraduate Medical Education and Research, Puducherry 605006, India; 4 Department of Psychiatry, Faculty of Medicine, University of Kelaniya, Kelaniya, Sri Lanka; 5 Department of Psychiatry, King George's Medical University, Lucknow-226003, UP, India; 6 Department of Graduate Nursing, Bangabandhu Sheikh Mujib Medical University, Dhaka, Bangladesh; 7 Department of Research - Transcultural Psychosocial Organization Nepal, Department of Community Medicine and Public Health - KIST Medical College, Kathmandu, Nepal

**Keywords:** Depression, non-fatal attempt, self-harm, South Asia, suicide

## Abstract

**Background:**

Estimates of depression in suicidal behavior in South Asia would help to formulate suicide prevention strategies in the region that hasn't been assessed yet.

**Objectives:**

We aimed to systematically assess the prevalence of depression in fatal and non-fatal attempts of suicide in eight South Asian countries.

**Methods:**

We searched Medline, Embase, and PsychINFO by specific search terms to identify articles assessing depression in fatal and non-fatal attempts of suicide in South Asian countries published between 2001 and 2020. Two separate meta-analyses were conducted for fatal and non-fatal attempts. Due to the high heterogeneity of studies (96–98%), random-effects models were used to calculate pooled prevalence rates.

**Results:**

A total of 38 studies was identified from five south Asian countries (India [27], Pakistan [6], Sri Lanka [3], Nepal [1], and Bangladesh [1]). The majority of studies (*n* = 27) were published after 2010. Twenty-two studies reported non-fatal attempts, and sixteen reported suicide. The prevalence of depression among non-fatal attempts ranged from 14% to 78% where the pooled prevalence rate was 32.7% [95% CI 26–39.3%]. The prevalence of depression among suicides ranged from 8% to 79% where the pooled prevalence estimate was 37.3% [95% CI 26.9–47.6%].

**Conclusions:**

This review revealed the pooled prevalence of depression among fatal and non-fatal suicidal attempts in South Asian countries, which seems to be lower when comparedto the Western countries. However, a cautious interpretation is warranted due to the heterogeneity of study methods, sample size, and measurement of depression.

## Introduction

Suicide is a global public health issue (World Health Organization, 2021). The World Health Organization (WHO) estimated that just over 700 000 deaths happened by suicide worldwide in 2019, representing an annual global age-standardized suicide rate of 9 per 100 000, being the fourth-leading cause of death among 15–29-year-olds (WHO, 2021). It is the end result of a complex interaction between several risk factors which may include biological, personal, social, psychological, cultural, and environmental factors, but psychiatric disorders are one of the most crucial risk factors (WHO, 2014; Zalsman *et al*., 2016; Arafat and Kabir, 2017). Estimations indicate that about 90% of people who die by suicide experience some form of psychiatric illness (Cavanagh *et al*., 2003; Milner *et al*., 2013; Cho *et al*., 2016; Zalsman *et al*., 2016; Arafat and Kabir, 2017). Among psychiatric disorders, depression is the most common risk factor for suicides (Malakouti *et al*., 2015; Arafat and Kabir, 2017).

South Asia is a suicide-dense area with a handful of studies assessing the relationship between depression and suicidal behavior. South Asia represents approximately one-quarter (23%) of the global population, and one-fifth of the mental health cases live here (Bishwajit *et al*., 2017). A recent systematic review conducted by Arafat and colleagues assessed psychiatric disorders from psychological autopsy studies in the WHO-South East Asia (SEA) region; however, the proportion of depression was not delineated (Arafat *et al*., 2022). Another systematic review conducted by Knipe *et al*. (2019) studied psychiatric morbidities in low-and-middle-income countries, which also lacked calculation of the prevalence of depression in suicidal behavior. One systematic review assessed depression in suicidal behavior in WHO-SEA countries that included papers from 1956 to 2016 (Ahmed *et al*., 2017). The review included articles published over a wide duration, but it assessed only 19 articles over the 60-year duration in the eleven countries of the WHO-SEA region, indicating a sporadic distribution of papers. Additionally, the authors considered the articles where depression was measured without any objective instruments in the same manner as articles where it was measured with objective instruments, even though this distinction was clearly stated. Also, a meta-analysis was not performed. Given such limitations in previous literature, we aimed to assess the prevalence of depression in suicidal behavior in the South Asian countries (Afghanistan, Bangladesh, Bhutan, India, Maldives, Nepal, Pakistan, and Sri Lanka) based on recent updates (2001–2020). We also aimed to estimate the pooled rates of depression among fatal and non-fatal suicidal attempts, and categorizing studies based on how depression was measured, viz. with/without objective instruments.

## Methods

### Search strategy

**Fig. 1.**
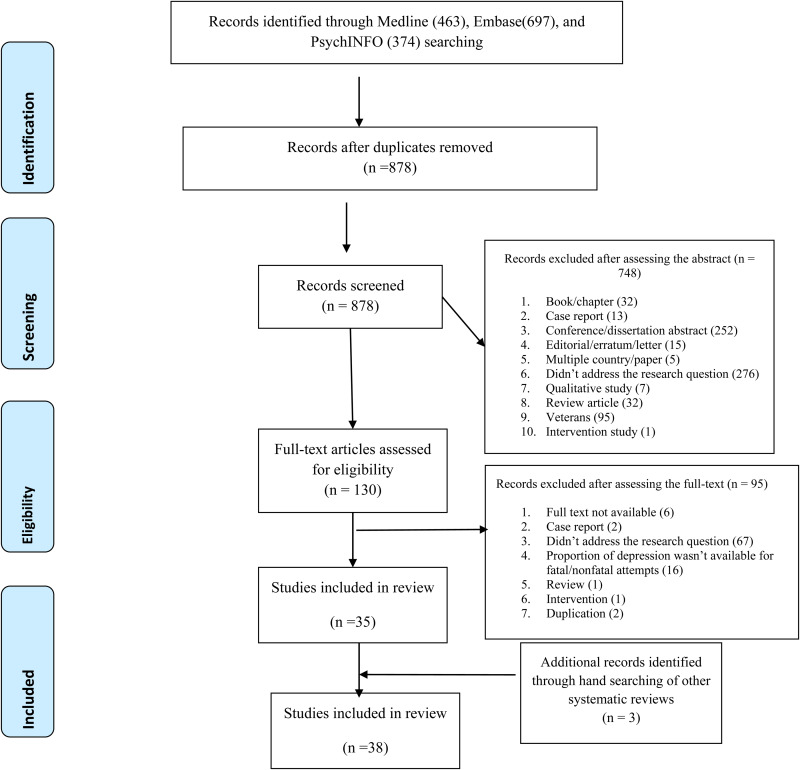
PRISMA 2009 flow diagram.

We searched Medline, Embase, and PsychINFO by specific search terms for the identification of articles assessing depression in suicidal behavior in South Asia. The details of the search strategy are mentioned in online Supplementary file 1 and the protocol was registered in advance (PROSPERO 2021 CRD42021285207). We included the articles published between 2001 and 2020 irrespective of the period of data collection. We also did a hand search of recently published systematic reviews to ensure the inclusion of a maximum number of articles (Ahmed *et al*., 2017; Knipe *et al*., 2019; Arafat *et al*., 2022). The stepwise search details are mentioned in Fig. 1. Two separate authors screened the articles independently and the third opinion was sought if any ambiguity appeared.

### Inclusion criteria

Original articles, studies with quantitative estimates, published in the English language, and articles available in full-text were included.

### Exclusion criteria

We excluded articles having samples from the veterans, and articles with qualitative outcomes. Articles from multiple country data and multiple articles from the same project were excluded. Any type of review, editorial, erratum, letters without primary data were excluded. Articles from media reports were excluded.

### Data extraction

The search identified 38 articles from five South Asian countries (Bangladesh [1], India [27], Nepal [1], Pakistan [6], and Sri Lanka [3]). Two authors extracted the data independently and a third author cross-checked the data extraction.

### Quality assessment

The methodological quality of each article included in the systematic review was assessed. Two authors independently evaluated the risk of bias for the research papers based on non-randomized studies. The tool applied for quality assessment was a modified version of the Newcastle-Ottawa Scale (NOS). The scale evaluated each article on the following aspects: (1) sample representativeness and size (2) comparability between respondents and non-respondents (3) ascertainment of depressive or suicidal symptoms (4) thoroughness of descriptive statistics reporting (Rotenstein *et al*., 2016). All components of the scale are to be summated to generate a total score for the risk of bias. The total score ranges from 0 to 5.The research studies were evaluated to be at low risk of bias (⩾3 points) or high risk of bias (<3 points).

### Data analysis

The effect size of interest in the present meta-analysis was the prevalence of depression. Pooled prevalence rates were calculated separately for fatal and non-fatal attempts. The Meta and Metafor packages in the R environment were used for the calculation of pooled effects, heterogeneity statistics, subgroup analysis, publication bias analysis, and sensitivity analysis. Heterogeneity among studies was examined using the *I*^2^ statistic and Cochran's*Q* test. Due to significant heterogeneity, random-effects models were used for the meta-analysis. Pooled estimates were calculated using the inverse variance method of random effects models andreported as proportions with 95% confidence intervals. Publication bias was examined by visual inspection of funnel plots, as well as the rank-correlation and Egger's tests for funnel plot asymmetry; a non-significant *p* value (>0.05) in the rank-correlation and Egger's tests indicates low publication bias. Subgroup analysis was performed by dividing the studies based on the use of standard methods for assessing depression. In subgroup analysis, a common between-study variance component was assumed due to the small number of studies in each subgroup, and tau-squared was pooled across the groups.

### Ethical aspects

We reviewed secondary data from publicly available articles. Therefore, no institutional review board approval was sought to conduct the study.

## Results

### Distribution of the studies

In this systematic review, 38 studies fulfilled the eligibility criteria (Fig. 1). The highest number of studies were identified from India(*n* = 27), followed by Pakistan (*n* = 6), Sri Lanka (*n* = 3), Bangladesh (*n* = 1), and Nepal (*n* = 1). The majority of studies (*n* = 27) were published after 2010. Out of the 38 studies, 22 reported on non-fatal attempts, and 16 reported on suicides.

### Characteristics of studies on non-fatal attempts

The characteristics of studies among non-fatal attempts are presented in Table 1. Studies from India (*n* = 17), Pakistan (*n* = 4), and Sri Lanka (*n* = 1) reported on depression among non-fatal attempts. The sample size of studies ranged from 27 to 1159 and the total number of non-fatal attempts in the 22 studies was 4597. The percentage of males in these samples ranged from 25% to 78%. Mean age was reported in only seven studies, and the age range was reported in six studies. Whether the study population was from a rural or urban area could be identified in 14 studies: five studies were based on rural populations, four studies were on urban population and five had mixed rural/urban populations (Table 1).

**Table 1.** Characteristics of studies of non-fatal attempts

Study no.	Study	Country	Place of study	Study design	Depression assessment method	Rural/Urban	Sample size	Male: Female ratio	Mean (s.d.) age, [range]	Prevalence of depression (%)	Risk of bias
1	Chandrasekaran and Gnanaselane (2005)	India	Pondicherry		MADRS		341	0.81:1	26.1 (±9.3)	31	Low
2	Kumar *et al*. (2006)	India	Pondicherry		MADRS	Rural	203	1.05:1	[16–65]	29.56	Low
3	Parkar *et al*. (2006)	India	Mumbai	Cross-sectional	SCID-I	Urban	196	1.1:1		43.9	Low
4	Syed and Khan (2008)	Pakistan	Karachi	retrospective descriptive study	Clinical diagnosis		69	0.56:1		18.8	Low
5	Bansal *et al*. (2011)	India	Panjab	Cross-sectional	ICD-10	Urban	100	1.56:1		46	Low
6	Bansal and Barman (2011)	India	Bathinda	Cross-sectional	ICD-10	Urban	100	0.72:1		36	Low
7	Yadiyal and Aruna (2011)	India	Mangalore	Cross-sectional	SCID-I		51	1.12:1	[18–65]	33.33	High
8	Vishnuvardhan and Saddichha (2012)	India	Bangalore		SCID-I		100	0.92:1		14	Low
9	Rao *et al*. (2013)	India		Cross-sectional	MINI plus	Rural and urban	100	1.08:1	27.31 (±8.68)	35	Low
10	Chowdhury *et al*. (2013)	India	Sundarban	Prospective study	SCID		89	0.34:1		14.6	Low
11	Tahir *et al*. (2013)	Pakistan	Mianwali	Population-based survey		Rural and urban	104	3.5:1		33	Low
12	Krishna *et al*. (2014)	India	Mysore	Cross-sectional	MINI	Rural	200	1.02:1	29.1 (±10.5) [18–70]	15.5	Low
13	Rajapakse *et al*. (2014)	Sri Lanka	Peradeniya		PHQ-9	Urban	949	0.79:1		51.1	Low
14	Kosaraju *et al*. (2015)	India	Hyderabad	Prospective study	Clinical diagnosis	Rural	36	0.8:1	[16–70]	38.9	High
15	Halder and Mahato (2016)	India	Kolkata	Cross-sectional	ICD-10	Urban, semi-urban and rural	100	0.38:1	23.56 (±6.38) [15–40]	41	Low
16	Husain *et al*. (2019)	Pakistan	Karachi		BDI		221	0.45:1		78.3	Low
17	Reuben *et al*. (2020)	India	Salem	Case–control	HDRS	Rural and urban	100	1.08:1	27.31 (±8.68)	60	Low
18	Khan and Ayub (2020)	Pakistan	Peshawar	Prospective study			102	0.61:1		14.8	Low
19	Kar (2010)	India	Orissa	Cross-sectional	ICD-10	Rural	149	0.77:1		24.8	Low
20	Kumar *et al*. (2015*b*, 2015*a*)	India	Kerala	Non-Experimental descriptive	ICD-10	Rural	1159	0.99:1	68.89 (±4.74) (older), 29.34 (±10.70) (younger)	24.42	Low
21	Ramdurg *et al*. (2011)	India	New Delhi	Prospective study	ICD-10	Rural and urban	27	1.25:1	31.5 (±11.62) [16–64]	19	Low
22	Sahoo *et al*. (2016)	India	Jamshedpur	Cross sectional			101	0.71:1		30.7	Low

MADRS, Montgomery-Asberg Depression Rating Scale; SCID, Structured Clinical Interview for DSM-IV Axis I Disorders; ICD, International Classification of Diseases; MINI, Mini-International Neuropsychiatric Interview; PHQ, Patient Health Questionnaire; BDI, Beck Depression Inventory; HDRS, Hamilton Depression Rating scale.

The use of a structured diagnostic interview or validated rating scale to assess depression was reported in 17 studies. Diagnostic interviews used International Classification of Diseases (ICD) diagnostic criteria (*n* = 6), Diagnostic and Statistical Manual ofMental Disorders (DSM) criteria (*n* = 4), and the Mini International Neuropsychiatric Interview (*n* = 2); in two studies, a clinical diagnostic interview had been used, but the exact type of criteria was not reported. Studies used several rating scales namely, Montgomery Asberg Depression Rating Scale (*n* = 2), Beck Depression Inventory (*n* = 1), Hamilton Depression Rating scale (*n* = 1) and Patient Health Questionnaire (PHQ-9) (*n* = 1). A standard method (structured interview or rating scale) was not reported in three studies.

### Prevalence of depression among non-fatal attempts

**Fig. 2.**
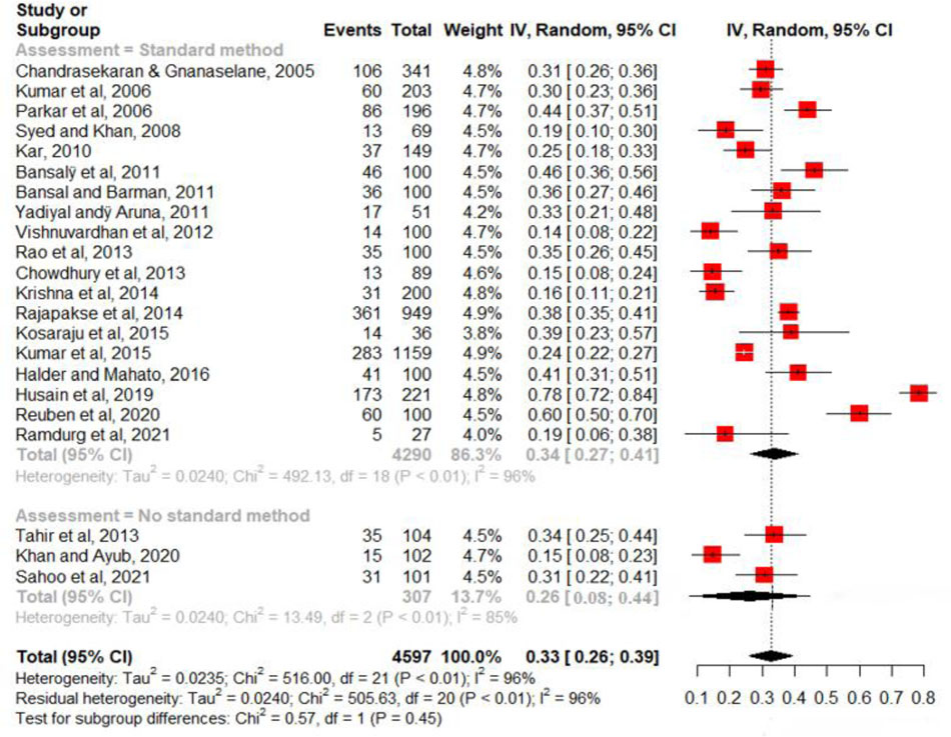
Pooled estimate for the prevalence of depression among non-fatal attempts of 22 studies.

The prevalence of depression among non-fatal attempts ranged from 14% to 78%. There was significant heterogeneity between the studies [*I*^2^ = 95.9% (94.8%; 96.8%) and Cochran's *Q* = 515.8, df = 21, *p* < 0.001]. Therefore, a random-effects model was used for estimating the pooled prevalence of depression. The pooled estimate for the prevalence of depression among non-fatal attempts was 32.7% [95% CI 26–39.3%] (Fig. 2).

A subgroup analysis was performed to test if the prevalence rate was different for studies that used a standard method of assessment and those that did not (Fig. 2). The pooled prevalence in studies that reported a standard method (*k* = 19) was 33.8%; and in studies that did not report a standard method (*k* = 3), the prevalence was 25.8%. This difference was, however, not statistically significant (*Q* = 0.64, df = 1, *p* = 0.42). A sensitivity analysis was performed by excluding studies with a high risk of bias (*n* = 2) from the meta-analysis; the pooled prevalence did not change remarkably [32.5% (95% CI 25.2–39.7%)].

### Characteristics of studies on suicide

The characteristics of studies on suicide are presented in Table 2. Studies from India (*n* = 10), Pakistan (*n* = 2), Sri Lanka (*n* = 2), Bangladesh (*n* = 1) and Nepal (*n* = 1) reported the presence of depression among people who died by suicide. The sample size of studies ranged from 27 to 857 and the total number of suicides in the 16 studies was 2509. The percentage of males in these samples ranged from 7.7% to 83%. Mean age was reported in six studies and age range was reported in seven studies. Whether the study population was from a rural or urban area could be identified in eight studies: One study was based on rural populations, two studies were on urban populations and five had mixed rural/urban populations. Nine studies reported using a standard diagnostic interview or validated rating scale to assess depression; a standard method was not reported in seven studies. Diagnostic interviews were based on ICD diagnostic criteria (*n* = 4) and DSM (*n* = 4). In one study, a rating scale (PHQ-9) had been used.

**Table 2.** Characteristics of studies of suicides

Study no.	Study	Country	Place of study	Study design	Depression assessment method	Rural/Urban	Sample size	Male: Female ratio	Mean (±s.d.) age, [range]	Prevalence of depression (%)	Risk of bias
1	Khan *et al*. (2005)	India		Case series psychological autopsy		Urban and rural	50	1.38:1	[15–35]	10	Low
2	Kanchan and Menezes (2008)	India	Manipal	Retrospective study		Rural	137	2.8:1	[16–82]	38.7	Low
3	Khan *et al*. (2008)	Pakistan	Karachi	Case–control psychological autopsy	ICD-10	Urban	100	4.88:1		79	Low
4	Abeyasinghe and Gunnell (2008)	Sri Lanka	Anuradhapura, Kurunegala; Hambantota	Case-series psychological autopsy	ICD-10		372	3.76:1	[10–94]	48.4	Low
5	Samaraweera *et al*. (2008)	Sri Lanka	Ratnapura	Case-series psychological autopsy	ICD-10		27	2.37:1	43 [15–74]	37	High
6	Chavan *et al*. (2008)	India	Chandigarh	Case series psychological autopsy		Rural, suburban, urban	101	1.34:1		11.9	Low
7	Manoranjitham *et al*. (2010)	India	Kaniyambadi Block, Vellore District,	Case–control psychological autopsy	SCID (DSM-III-R)	Rural	100	1.43:1	42.24 (±20.69)	2	Low
8	Srivastava (2013)	India	Goa	Case series psychological autopsy	ICD-10	urban, rural	100	2.33:1		54	Low
9	Tanna *et al*. (2013)	India	Gujarat	Retrospective study			110	1.07:1		8	Low
10	Kumar *et al*. (2015*b*, 2015*a*)	India	Lucknow	Retrospective study			857	0.08:1	33.74 (±11.64)	28.57	Low
11	Bhise and Behere (2016)	India	Wardha	Case–control psychological autopsy	SCID-I (DSM-IV-TR)		98	8.8:1		37.7	Low
12	Hagaman *et al*. (2017)	Nepal	Jumla & Kathmandu	Mixed method psychological autopsy study	PHQ-9	Rural and urban	39	1.16:1	32.9 (±17.55) [14–79]	38.5	Low
13	Sandeep *et al*. (2017)	India	Bhopal	Retrospective study		Rural and urban	200	1.56:1		39.5	Low
14	Abdullah *et al*. (2018)	Pakistan	Khyber Pakhtunkhwa	Case series psychological autopsy	DSM-5 criteria		63	1.52:1	22.1 (±3.08)	60.3	Low
15	Narayanan *et al*. (2020)	India	Kerala	cross-sectional			55		[10–19]	50.96	High
16	Arafat *et al*. (2021*b*, 2021*a*)	Bangladesh	Dhaka	Case–control psychological autopsy	SCID-I	Urban	100	0.96:1	26.30 (±12.36) [9–75]	44	Low

SCID, Structured Clinical Interview for DSM-IV Axis I Disorders; ICD, International Classification of Diseases; PHQ, Patient Health Questionnaire; DSM, Diagnostic Statistic Manual.

### Prevalence of depression among suicide

**Fig. 3.**
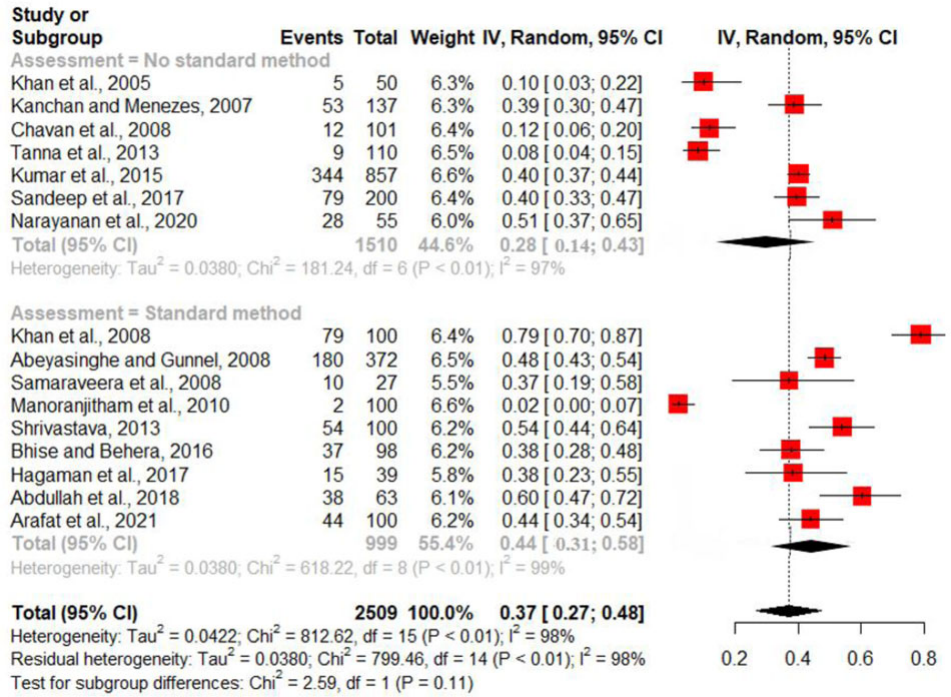
Pooled estimate for the prevalence of depression among suicides of 16 studies.

The prevalence of depression among suicides in individual studies ranged from 8% to 79%. There was significant heterogeneity between the studies [*I*^2^ = 98.2% (97.7%; 98.5%) and Cochran's *Q* = 812.62, df = 15, *p* < 0.0001]. Therefore, a random-effects model was used for estimating the pooled prevalence of depression. The pooled estimate for the prevalence of depression among suicide attempters was 37.3% [95% CI 26.9–47.6%] (Fig. 3).

A subgroup analysis was performed to test if the prevalence rate was different for studies that used a standard method of assessment and those that did not (Fig. 3). The pooled prevalence in studies that reported a standard method (*k* = 9) was 44.5%; and in studies that did not report a standard method (*k* = 7), the prevalence was 28.2%. This difference was not statistically significant (*Q* = 2.76, df = 1, *p* = 0.097).A sensitivity analysis was performed by excluding studies with a high risk of bias (*n* = 2) from the meta-analysis; the pooled prevalence did not change remarkably [36.3% (95% CI 24.8–47.9%)].

### Publication bias

**Fig. 4.**
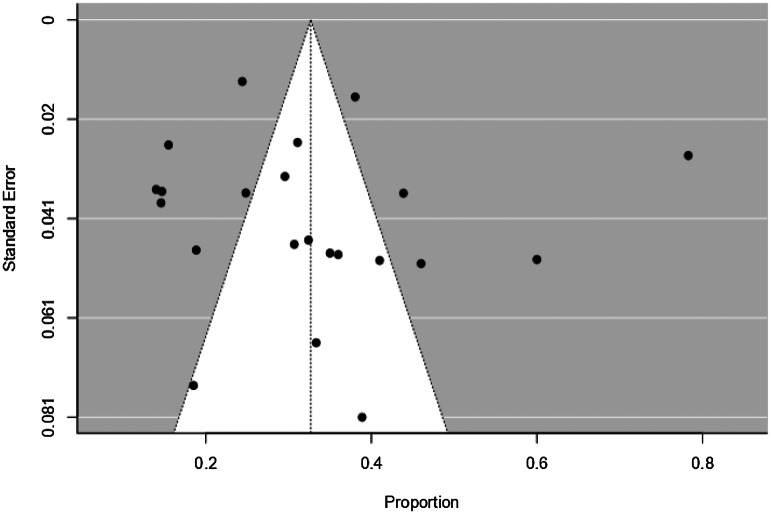
Funnel plot for studies on non-fatal attempts of 22 studies.

**Fig. 5.**
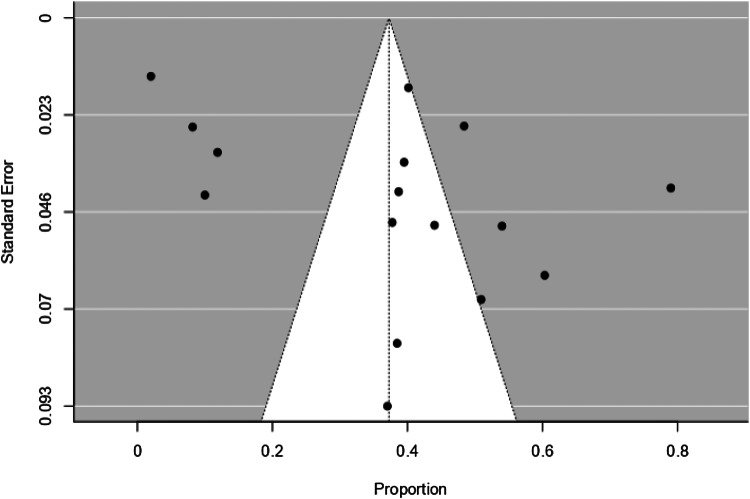
Funnel plot for studies on suicide.

The funnel plot for studies on non-fatal attempts is shown in Fig. 4. Egger's test of funnel plot asymmetry (*Z* = 0.189, *p* = 0.85) and rank correlation test (Kendall's tau = 0.264, *p* = 0.091) both indicate that the funnel plot is not significantly asymmetrical, suggesting low publication bias for the studies. The funnel plot for studies on suicides is shown in Fig. 5. Egger's test of funnel plot asymmetry (*Z* = 1.46, *p* = 0.145) and rank correlation test (Kendall's tau = 0.017, *p* = 0.965) both indicate that the funnel plot is not significantly asymmetrical, suggesting low publication bias for the studies.

As per the modified Newcastle Ottawa Quality assessment scale, four studies were found to have a high risk of bias (score < 3) and thirty-four were at low risk (score ⩾ 3). Among the reviewed studies, the Newcastle Ottawa Quality assessment scale score was between 0 and 5.Eight studies (*n* = 8, 20%) had high methodological quality, while the rest were of moderate (*n* = 19, 47.5%) and low quality (*n* = 13, 32.5%).

## Discussion

### Major findings of the review

South Asia is a suicide-dense region having a higher rate of suicide than the global average (WHO, 2021). Additionally, the role of psychiatric disorder as a risk factor has been under-studied in the region, albeit, the prevalence of the mental disorder among suicides varies from the West (Arafat *et al*., 2022). We aimed to assess depression among the fatal and non-fatal attempts of suicide in South Asian countries over the last two decades to see the recent scenario based on a search in Medline, Embase, and PsychINFO. To our knowledge, no prior systematic review has focused on estimating the prevalence of depression among those with suicidal behavior in South Asia. Our main findings revealed that the prevalence of depression among non-fatal attempts in 22 eligible studies ranged from 14% to 78%; and the corresponding figures for depression among suicides in 16 eligible studies ranged from 8% to 79%. The pooled prevalence estimate for depression among those with non-fatal attempts and suicide decedents was 32.7% and 37.3%, respectively. Findings from sub-group analysis revealed a relatively higher prevalence of depression in those studies where standard instruments were used, compared to studies where no standard instruments were utilized. This may indicate a possible under-reporting of depressive symptoms, and possibly, a higher stigma toward both psychiatric disorder and suicidal behavior. However, these subgroup differences did not reach statistical significance, likely due to the small number of studies in subgroups.

Ahmed *et al*. (2017), in their systematic review from eleven WHO-SEA regions, noted a wide range in prevalence rates of depression among suicides (6.9–51.7%) and non-fatal attempts (22–59.7%) similar to our findings. However, no meta-analysis was performed. Additionally, the authors searched databases through 60 years (1956–2016) and identified only 19 articles, whereas our study revealed recent estimates over the last 20 years from a higher number of studies and pooled the data in a meta-analysis. A previous review on psychiatric morbidity among patients with self-harm behavior revealed a pooled prevalence rate of 49.4% for depression in this group, which was the highest among all mental disorders (Hawton *et al*., 2013); our figures are relatively lower (32.7%). This difference may indicate a lower prevalence of depression in South Asian countries compared to the West among patients presenting with self-harm. On a similar note, authors who focused on synthesizing evidence from low- and middle-income countries found that mood disorders (including all ICD-10 mood disorder categories) were the most prevalent psychiatric disorder in both fatal and non-fatal suicidal behavior; the pooled prevalence estimates were 25 and 21%, respectively (Knipe *et al*., 2019). Nevertheless, separate pooled estimates for depression were not computed in that review. Another recent review of psychological autopsy studies also revealed a lower proportion of psychiatric disorders among suicides in the five WHO-SEA countries (Arafat *et al*., 2022). Two other reviews of psychological autopsy studies are available; one of them noted that depression was the most common mental illness among those who died by suicide but did not provide separate prevalence estimates for depression (Cavanagh *et al*., 2003). The other was a larger review that investigated geographical variations in the prevalence of psychiatric morbidity in suicide (Cho *et al*., 2016). Here, the authors reported a 51% prevalence rate for depressive disorder (including major depression and dysthymia) among suicide decedents; these figures are higher than what we have obtained, and the variations are likely due to geographical and methodological variations in studies. Most of these reviews primarily included research from high-income countries where mental illness is a major vulnerability factor as well as a driver for suicide.

Only four (Samaraweera *et al*., 2008; Yadiyal and Aruna, 2011; Kosaraju *et al*., 2015; Narayanan *et al*., 2020) of the 38 studies included in this review had a high risk of bias according to the assessment tool. The majority of the studies on non-fatal attempts (17 out of 22) used a structured assessment tool; in contrast, only 9 among 16 studies on suicide used this approach. The use of a structured assessment tool may be superior to unstructured interviews for generating a psychiatric diagnosis; this may be of particular relevance to the South Asian region, which is dominated by low- and middle-income countries where there is a considerable treatment gap for mental illness and psychiatric disorders are, often, under-diagnosed and undertreated (Naveed *et al*., 2020). This is also reflected in the quality assessment results, where many studies showed bias with regard to the ascertainment of exposure.

Significant heterogeneity (96–98%) was noted among study results, with wide-ranging prevalence estimates of depression among suicides and attempted suicide. Similar rates of heterogeneity have been noted in prior systematic reviews that investigated similar research questions (Hawton *et al*., 2013; Knipe *et al*., 2019). These differences can be likely attributed to two factors. Firstly, they may reflect real differences in depression-related morbidity among those exhibiting suicidal behavior. Secondly, they may reflect differences in the way the study was designed, conducted, interpreted, and analyzed. A deeper examination of the numbers suggests that the latter possibility may be relevant because Indian studies dominated the included literature and wide within-country variations were noted in prevalence rates. Variations between studies in definitions used for suicidal behavior may also have contributed to the observed heterogeneity. Future studies should use standardized instruments, recruit consecutive cases, and use standard definitions for defining cases to enhance the comparability of findings.

Our findings have important implications for suicide prevention in the region. In high-income countries, where a higher prevalence of depression is seen among suicide decedents (Cho *et al*., 2016), it follows that suicide prevention efforts need to focus on strengthening clinical services and care delivery bundles (Arafat *et al*., 2022). However, in the South Asian region, with a lower prevalence of depression, prevention of suicide must focus on a range of non-clinical contributory factors. These may include socio-economic determinants of suicide such as poverty (Iemmi *et al*., 2016; Arafat *et al*., 2021*b*, 2021*a*), gender inequality (Devries *et al*., 2011), and financial insecurity (Knipe *et al*., 2015). Targeting these factors will require a community-based suicide prevention approach with a multimodal focus: adequate insurance and welfare measures for alleviating poverty and improving employment, restricting access to lethal means, improving media portrayal of suicide, and utilization of community resources to enhance social connectedness are some of the potentially beneficial strategies. The latter strategy of increasing civic participation to link service providers to the needy has shown encouraging results in Japan and may be trialed elsewhere (Motohashi *et al*., 2007; CSRP Newsletter, 2009). On the other hand, the diagnosis of depression could be under-reported in suicidal behavior due to criminal status (Afghanistan, Bangladesh, Pakistan), stigma, low mental health literacy, poor mental health services coverage among suicides, a lower proportion of mental health personnel (Arafat *et al*., 2021*b*, 2022). At a policy level, given the relatively lower burden of depression among both attempters and those who died by suicide, suicide prevention efforts in South Asia may need to focus on locally relevant socio-economic stressors (such as poverty, unemployment), societal factors (such as rapid urbanization), and their interaction with depression and psychiatric morbidity. Such an approach will assist in modifying various factors in the causal pathway to suicide in these settings. Local and regional mental health advocacy groups like SAARC Psychiatric Federation could lead the countries to create awareness and reduce stigma related to suicide and depression (mental illness), further investigations to identify whether any under-reporting of depression would be benefitted.

### Strength and limitations of the study

This is the first systematic review assessing depression and suicidal behavior in South Asia during the immediate past two decades and creating pooled prevalence rate for depression. We searched three major databases (Medline, Embase, and PsychINFO). However, several limitations should be considered while generalizing the study results. Firstly, there is heterogeneity in the study methods, sample size, sampling frame and instruments that may affect the prevalence of depression from context to context. Secondly, the majority of the studies (27/38) were identified from India which would also affect the generalizability of the results.

## Conclusions

This review revealed the pooled prevalence of depression among fatal and non-fatal suicidal attempts in five South Asian countries, which seems to be lower when compared to the Western countries. Additionally, a relatively higher prevalence of depression was noted among studies using standard instruments indicating possible underreporting and stigma. A cautious interpretation of these findings is warranted due to substantial methodological and statistical heterogeneity noted in the analysis. Nevertheless, our findings have important implications for suicide research and prevention efforts in the region; most importantly, it highlights the need for a multimodal approach to suicide prevention that targets a range of contributory factors apart from mental health morbidity.

## Data Availability

The data that support the findings of this study are available on request from the corresponding author.

## Supplementary material

For supplementary material accompanying this paper visit https://doi.org/10.1017/gmh.2022.20. click here to view supplementary material
