# Supplementary material for: Depression and suicidal behavior in South Asia: a systematic review and meta-analysis
Source: Glob Ment Health (Camb). 2022 Apr 1;9:181–92. doi: 10.1017/gmh.2022.20 (PMC9806989; doi:10.1017/gmh.2022.20)
Supplement: Supplementary file 1 [file S2054425122000206sup001.docx]

**Supplementary file 1:** Search details

**Ovid MEDLINE(R) <1946 to present>**

1 (depression or (depressive and disorder) or (mood and disorder) or (mental and disorder) or (mental and illness) or (psychiatr* and disorder) or (psychiatr* and illness) or (psychological and autopsy)).ab. or (depression or (depressive and disorder) or (mood and disorder) or (mental and disorder) or (mental and illness) or (psychiatr* and disorder) or (psychiatr* and illness) or (psychological and autopsy)).ti. 478228

2 (self?harm* or suicid*).ab. or (self?harm* or suicid*).ti. 82107

3 (Afghanistan* or Bangladesh* or Bhutan* or India* or Maldiv* or Nepal* or Pakistan* or Sri Lanka*).ab. or (Afghanistan* or Bangladesh* or Bhutan* or India* or Maldiv* or Nepal* or Pakistan* or Sri Lanka*).ti. 231616

4 1 and 2 and 3 568

5 limit 4 to (english language and yr="2001 - 2020") 463

**Embase <1980 to 2021 Week 39>**

1 (depression or (depressive and disorder) or (mood and disorder) or (mental and disorder) or (mental and illness) or (psychiatr* and disorder) or (psychiatr* and illness) or (psychological and autopsy)).ab. or (depression or (depressive and disorder) or (mood and disorder) or (mental and disorder) or (mental and illness) or (psychiatr* and disorder) or (psychiatr* and illness) or (psychological and autopsy)).ti. 663358

2 (self?harm* or suicid*).ab. or (self?harm* or suicid*).ti. 103154

3 (Afghanistan* or Bangladesh* or Bhutan* or India* or Maldiv* or Nepal* or Pakistan* or Sri Lanka*).ab. or (Afghanistan* or Bangladesh* or Bhutan* or India* or Maldiv* or Nepal* or Pakistan* or Sri Lanka*).ti. 306157

4 1 and 2 and 3 811

5 limit 4 to (english language and yr="2001 - 2020") 697

**APA PsycInfo <1806 to September Week 4 2021>**

**1** (depression or (depressive and disorder) or (mood and disorder) or (mental and disorder) or (mental and illness) or (psychiatr* and disorder) or (psychiatr* and illness) or (psychological and autopsy)).ab. or (depression or (depressive and disorder) or (mood and disorder) or (mental and disorder) or (mental and illness) or (psychiatr* and disorder) or (psychiatr* and illness) or (psychological and autopsy)).ti. 395785

2 (self?harm* or suicid*).ab. or (self?harm* or suicid*).ti. 68501

3 (Afghanistan* or Bangladesh* or Bhutan* or India* or Maldiv* or Nepal* or Pakistan* or Sri Lanka*).ab. or (Afghanistan* or Bangladesh* or Bhutan* or India* or Maldiv* or Nepal* or Pakistan* or Sri Lanka*).ti. 47057

4 1 and 2 and 3 474

5 limit 4 to (english language and yr="2001 - 2020") 374
